# How to optimize the use of MRI in anatomic ACL reconstruction

**DOI:** 10.1007/s00167-012-2153-9

**Published:** 2012-08-15

**Authors:** Paulo Araujo, Carola F. van Eck, Maha Torabi, Freddie H. Fu

**Affiliations:** 1 Department of Orthopaedic Surgery, University of Pittsburgh Medical Center, Kaufman Building Suite 1011, 3471 Fifth Avenue, Pittsburgh, PA 15213 USA; 2 Department of Radiology, University of Pittsburgh Medical Center, Pittsburgh, USA

**Keywords:** Anatomic ACL reconstruction, MRI measurements, ACL inclination angle, ACL length, ACL insertion site, Graft size

## Abstract

**Purpose:**

Magnetic resonance imaging (MRI) is the most current diagnostic imaging procedure for suspected ACL injuries. It is an accurate, highly sensitive and specific tool for the diagnosis of ACL tears, graft tears and associated injuries. However, it can also be used for various other aspects of anatomic ACL reconstruction.

**Methods:**

Special sequences as the oblique sagittal plane should be obtained from a parallel line to the lateral epicondyle, ensuring a proper visualization of both bundles of the ACL. Another special set of images, the oblique-coronal sequence, allows for the ACL long-axis evaluation. The coronal-oblique sequence increases the sensitivity and specificity of diagnosing isolated AM or PL bundle injuries and also helps to visualize the proximal insertion of the bundles for haemorrhage and rupture.

**Results:**

Quantitative measurements can be taken from a proper MRI protocol, so as to determine the rupture pattern; measure insertion site size, inclination angle and autograft size; and evaluate for post-operative complications. These parameters help surgeons to objectively decide for a better graft and technique for an individualized approach and to evaluate the anatomic placement of the graft.

**Conclusions:**

MRI can be used in different ways, serving as a very valuable tool in anatomic ACL reconstruction. Special protocols can provide accurate visualization of the double-bundle anatomy. Objective parameters to aid in pre-operative decisions and graft’s anatomic placement evaluation can be also extracted from the MR images.

## Introduction

The goal of anatomic anterior cruciate ligament (ACL) reconstruction is the restoration of the ACL to its native dimensions, collagen orientation and insertion sites [29]. The ACL consists of two functional bundle, the anteromedial (AM) and posterolateral bundle (PL), which together provide both anterior and rotatory stability of the knee.

Magnetic resonance imaging (MRI) is the most frequent diagnostic imaging procedure for suspected ACL injuries. It is an accurate, highly sensitive and specific tool for the diagnosis of ACL tears, graft tears and associated injuries [7, 17]. The application of MRI in anatomic ACL reconstruction is rapidly expanding. Currently, MRI is used not only for diagnosis, but also for the measurement of the size and inclination angle of the ACL, pre-operative assessment of the size of autograft sources and post-operative evaluation of graft healing [1, 15]. MRI has also been used in several other evaluations related to ACL reconstruction, such as tunnel enlargement [25], roof impingement [13], tibiofemoral relation [27] and cartilage degenerative changes after ACL injury [18]. This paper focuses on all applications of MRI in anatomic ACL reconstruction. It will also describe an MRI ACL protocol for ACL injury and reconstruction.

## Protocol for MR imaging of the ACL

A 1.5-T magnet is used with an open-bore configuration (Magnetom Espree, Siemens Medical Solutions, Malvern, PA, USA). The ACL is optimally imaged using multiple planes. The different pulse sequences used for evaluating the ACL are outlined in Table 1, including the role of each sequence. The normal ACL on MRI has a hypo-intense appearance on T1- and T2-weighted sequences.

**Table 1:** Routine pulse sequences performed for assessment of the ACL

Sequence	Field of view (mm)	Slice thickness (mm)	Function
Axial T2 TSE FS	140	4, skip1	Identify fluid in tunnel, identify disruption of graft fibres
Sagittal PD TSE	140	3, skip 1	Assess collagen maturation of graft
Sagittal T2 TSE FS	140	3, skip 1	Identify fluid in tunnel, identify disruption of graft fibres
Coronal T1 SE	140	3, skip 1	Assess collagen maturation of graft, evaluate associated bone injury
Coronal T2 TSE FS	140	4, skip 2	Identify fluid in tunnel, identify disruption of graft fibres
Coronal-oblique TSE PD	120	2.5, skip 0.3	Assess collagen maturation of graft, identify disruption of graft fibres
Sagittal 3D SPACE	150	2.5, skip 0.3	High resolution, thin section images for optimal anatomic evaluation of AM and PL bundles

**Fig. 1:**
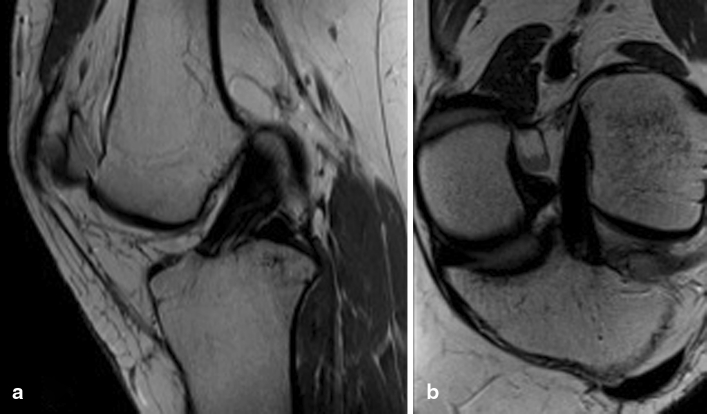
**a** Sagittal oblique view of the intact ACL. **b** Coronal-oblique view of the intact ACL

The protocol starts with a scout image in the axial, sagittal and coronal plane. The axial images are true axial and are derived directly from the scout images. The sagittal images are based on anatomic landmarks to individualize the sequence to ensure proper visualization of the ACL and its two bundles for every patient. A plane is prescribed along the lateral femoral epicondyle at the level of the lateral collateral ligament (LCL), which is a constant anatomic landmark with little interpersonal variability. An oblique sagittal sequence is also obtained, which runs parallel to the ACL and optimally visualizes both the AM and PL bundles (Fig. 1). Routine coronal images are then prescribed, followed by a special oblique-coronal sequence (Fig. 1). This plane is in the long axis of the ACL, starting at the intercondylar roof of Blumensaat’s line. The coronal-oblique sequence increases the sensitivity and specificity of diagnosing isolated AM or PL bundle injuries [8, 14]. It also helps to visualize the proximal insertion of the bundles for haemorrhage and rupture. A slice thickness of 2.5 mm can differentiate the two bundles as separate entities or alternative [6]. 3D imaging can also be obtained with the same 1.5-T magnet.

## Evaluation of ACL rupture

**Fig. 2:**
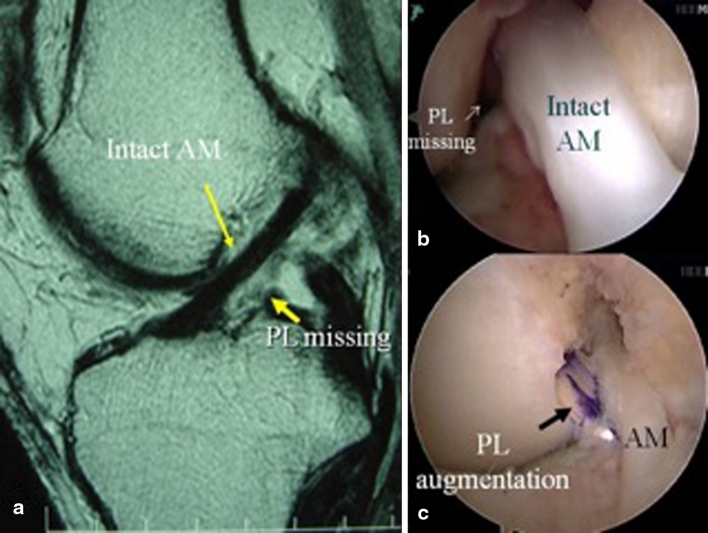
**a** MRI showing absence of PL bundle with AM bundle intact. **b** Arthroscopic picture confirming the absence of the PL bundle. **c** PL bundle augmentation

**Fig. 3:**
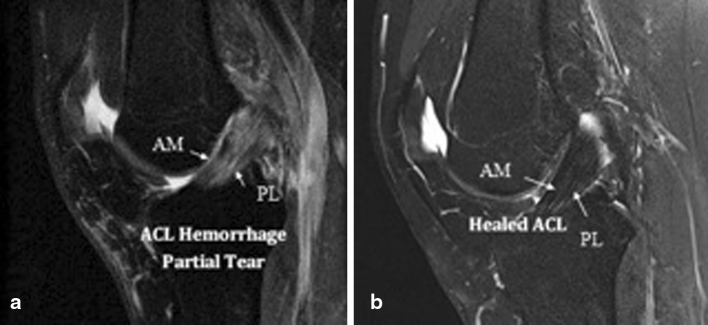
**a** MRI showing AM bundle isolated tear. **b** Six months later, MRI showing complete healing

The ACL rupture can be clinically diagnosed most of the time. With the high sensitivity and specificity of physical examination, most ACL ruptures can be diagnosed without the need for further imaging [4]. However, in cases where the physical examination is inconclusive, MRI can provide high degree of diagnostic accuracy [7, 17, 28]. The physical examination may especially be unclear in the case of isolated bundle ruptures [31]. When there is a large Lachman, but only a gliding or no pivot shift, an isolated AM bundle rupture should be expected [19, 20]. Similarly, when there is a large pivot shift, with minimal anterior displacement on Lachman examination, an isolated PL bundle is likely [19, 20]. Confirmation of this diagnosis pre-operative on MRI is essential for planning of treatment (Fig. 2). Partial ACL tears may heal or progress, which can be carefully followed using MRI (Fig. 3). In addition, symptomatic partial ACL tears are suitable to undergo augmentation surgery [5, 21, 24, 26, 30].

## Measuring ACL insertion site size and ACL length

**Fig. 4:**
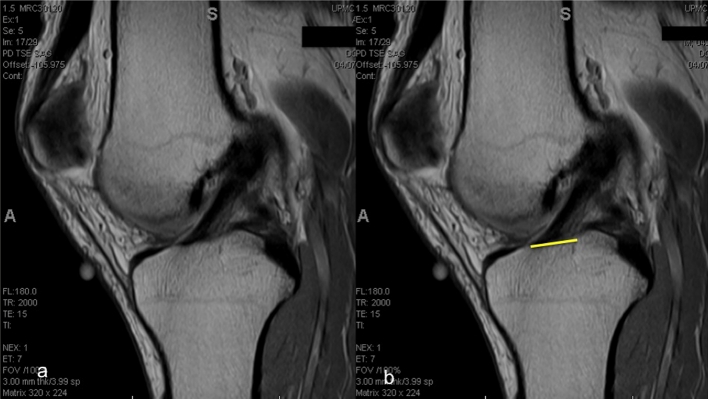
**a** Sagittal MRI cut best showing the ACL attachment to the tibia; **b** the most anterior and most posterior fibres attaching the tibia are connected by a line that represents the insertion site size

**Fig. 5:**
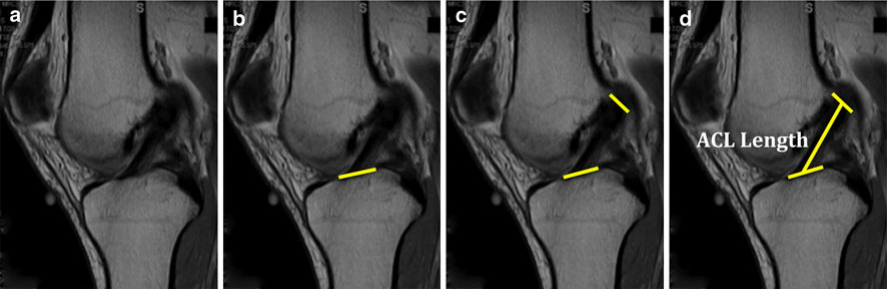
**a** MRI sagittal cut best showing tibial and femoral insertion sites is chosen; **b** tibial insertion site is highlighted; **c** femoral inserion site is highlighted; **d** the distance between the mid-portion of the tibial and femoral insertion sites are connected and measured to know ACL length

The size of the ACL varies greatly amongst individuals [9, 10, 15, 22, 23]. In a clinical study of 137 patients undergoing ACL reconstruction, the tibial ACL insertion site measured 17 mm in length with a standard deviation of 2 mm, while the femoral insertion site measured 16.5 mm in length with a standard deviation of 2 mm [15]. One of the principles of anatomic ACL reconstruction is to restore the native ACL insertion site size and dimensions. This means that a patient’s native ACL insertion site size determines the type of procedure (single- or double-bundle reconstruction), tunnel size and graft size [20]. The tibial insertion site can be measured pre-operatively, which facilitates pre-operative planning (Fig. 4). A single sagittal proton density image best showing the ACL fibre attachment to the tibia is selected. The image is then imported to OSIRIX (Version 3.7.1., Pixmeo Sari, Bernex, Switzerland) for analysis. The most anterior and the most posterior portion of the ACL attachment is marked, and the distance between them is measured. In addition, the length of the native ACL can be measured using the same steps described for the tibial insertion site measurement (Fig. 5). The clearest MRI cut showing both the tibial and femoral insertion sites is chosen, and the distance between the mid-portion of the insertion sites is measured. Determining ACL size may facilitate preoperative planning for an individualized approach to ACL reconstruction in terms of graft options, as well as determining whether the patient will undergo single- or double-bundle surgery. Post-operatively, the reconstructed ACL insertion size can be compared to the native ACL insertion site size to see how much of the ACL has been restored. The same is possible for the intra-articular graft length.

## ACL inclination angle

**Fig. 6:**
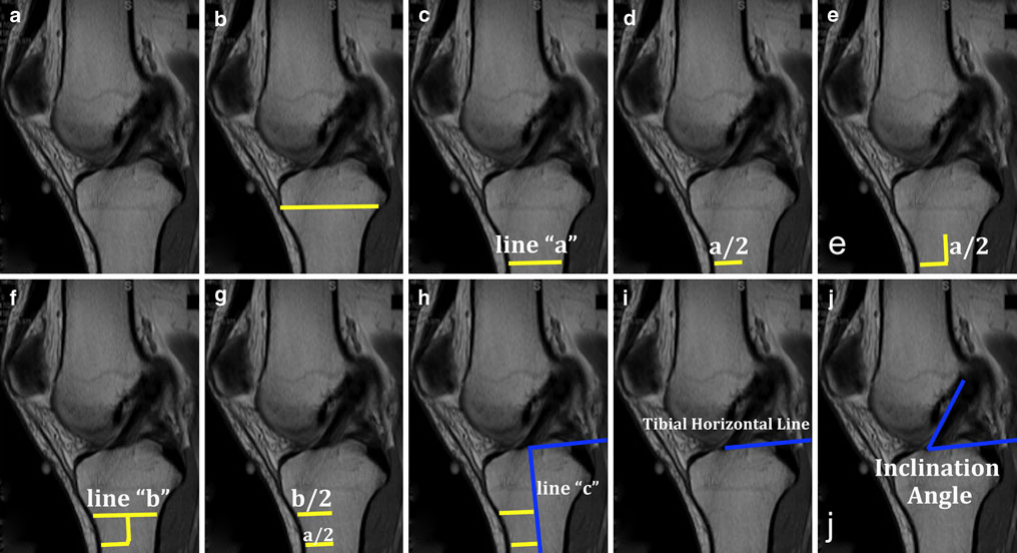
**a** Sagittal MRI showing the ACL and physeal scar; **b** line drawn over the physeal scar; **c** *Line* a, parallel to the physeal scar; **d** half of the *line* a; **e** and **f** *Line* b is drawn proximally to the *line* a in half of its distance; **g** and **h** half of the distance of *Lines* a and b are used as parameter to create *Line c* representing the long axis of the tibia; **i** tibial *Horizontal Line*; **j**. inclination Angle of the ACL

The inclination angle is a simple and easy measurement that can be used for evaluation of graft placement [1, 3, 11]. Measurement of the inclination angle is performed on the sagittal sequence (Fig. 6). The long axis of the tibia is estimated by selecting its most distal portion available in the image. The width of the diaphysis (line a) parallel to the physeal scar is then measured. Proximally in the diaphysis, another width measurement (line b) is taken half of the distance from the most distal width. The mid-points of these lines are connected coursing the long axis of the tibia (line c) and a line perpendicular to the line c, named the tibial horizontal line (THL), is drawn. A parallel line to the THL is created and its end placed at the most anterior portion of the ACL. The angle between this line and the most anterior fibres of the ACL is designated as the ACL inclination angle. Normal ACL inclination angle ranges from 43° to 57° [12]. This method, described by Illingworth et al. [12], provides an intra-observer and interobserver reliability of (ICC) 0.85 (95 % CI .73–.92) and 0.75 (95 % CI .60–.85) for native MRI inclination angle measurements and 0.98 (95 % CI .96–.99) and 0.91 (95 % CI .85–.95) for SB ACL reconstructions, respectively. The sensitivity and specificity, using a ROC curve for anatomic determination, for an inclination angle of 55.0°, were 100 and 87.5 %, respectively. Before the surgery, the native inclination angle can be measured; however, when the ACL is torn, this measurement becomes less accurate. After anatomic single- or double-bundle ACL reconstruction, the inclination angle of the reconstructed ACL can be compared to that of the native ACL. When the ACL is truly reconstructed in an anatomic fashion, the pre- and post-operative inclination angle should be within a few degrees of each other.

This principle can also be applied to revision surgery. The inclination angle can be used to determine whether the previous graft was placed anatomically, by comparing the inclination angle with the ACL inclination of the contralateral limb. If there is a large discrepancy, this means the ACL was most likely placed non-anatomically and use of the existing tunnels during revision surgery should be avoided [30]. After revision surgery, the inclination angle of the graft can be re-measured, to ensure whether satisfactory restoration of the native inclination angle was obtained. The inclination is a simple but reliable method, which is highly correlated with the degree of anatomic tunnel placement [12].

## Pre-operative graft size of BPTB and quadriceps tendon autograft

**Fig. 7:**
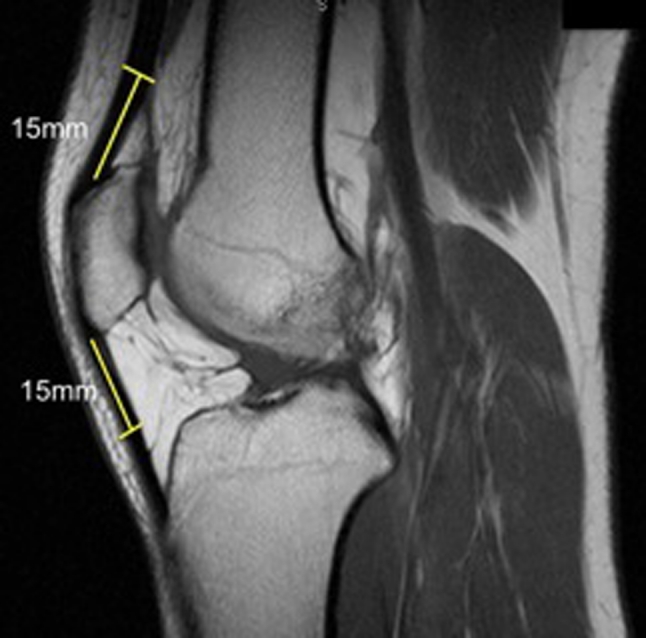
The quadriceps and patellar tendon thicknesses are measured 15 mm proximal and distal, respectively, from the patellar poles

Many graft options exist for ACL reconstruction. Both allograft and autograft material can be used. This decision is based on patient age, activity level, concomitant injuries, previous surgeries, and patient and surgeon preference. Allograft material offers less anaesthesia time, no additional skin incisions, less post-operative pain and free choice of graft diameter and length. Autograft has the benefit of low risk of disease transmission and faster graft healing [16]. The disadvantage of autograft material is that the graft size depends on the size of the donor tissue. However, for bone-patellar tendon-bone (BPTB) and quadriceps tendon autograft, it is possible to evaluate graft size pre-operatively on MRI. On the sagittal proton-density MRI sequence, both tendons can be visualized and their thickness can be assessed (Fig. 7). The slice that demonstrates maximum anterior-to-posterior (AP) thickness of the quadriceps tendon is selected. A line of 15 mm starting from the mid-portion of the superior pole of the patella following quadriceps tendon proximally is drawn. Perpendicular to this line, another line connecting the most anterior to the most posterior portion of the tendon represents the thickness of the quadriceps tendon. The same method is used to determine the patellar tendon thickness using the distal pole of the patella as a starting point.

When the thickness of the tendon is less than 7 mm, there may not be sufficient graft for a double-bundle reconstruction and other graft options including hamstring autograft and allograft should be discussed with the patient.

## Evaluation of the graft after anatomic ACL reconstruction

MRI can also be used for post-operative evaluation after anatomic single- or double-bundle ACL reconstructions. When anatomic double-bundle reconstruction has been performed, the AM and PL bundle can be visualized, evaluated and measured separately. Post-operative MRI evaluation can consist of measuring the size of the restored insertion site and intra-articular graft length.

Recently, there has been an increased use of MRI for evaluation of graft healing. Although much remains unknown about the exact time line of ACL healing on MRI, some global stages can be observed, indicated by variation in signal intensity [2]. The graft remodels between 4 and 8 months after surgery [2]. During this period, revascularization of the neoligament, as well as re-synovialization, takes places. These processes increase the signal intensity [2]. After this initial period, the ACL graft will return to a hypo-intense signal, comparable to the native ACL, which will take approximately 1–2 years [2]. However, this process can differ depending on what graft source is used, for example allograft or autograft material [16].

## Conclusion

In conclusion, MRI is very valuable in anatomic ACL reconstruction. Special protocols ensure accurate visualization of the double-bundle anatomy and individual evaluation of the AM and PL bundles. MRI can be used to determine the rupture pattern; measure insertion site size, inclination angle, ACL and graft length, and autograft size; and assess for post-operative complications.

## References

[1] Ahn JH, Lee SH, Yoo JC, Ha HC (2007). Measurement of the graft angles for the anterior cruciate ligament reconstruction with transtibial technique using postoperative magnetic resonance imaging in comparative study. Knee Surg Sports Traumatol Arthrosc.

[2] Amiel D, Kleiner JB, Roux RD, Harwood FL, Akeson WH (1986). The phenomenon of “ligamentization”: anterior cruciate ligament reconstruction with autogenous patellar tendon. J Orthop Res.

[3] Ayerza MA, Múscolo DL, Costa-Paz M, Makino A, Rondón L (2003). Comparison of sagittal obliquity of the reconstructed anterior cruciate ligament with native anterior cruciate ligament using magnetic resonance imaging. Arthroscopy.

[4] Benjaminse A, Gokeler A, van der Schans CP (2006). Clinical diagnosis of an anterior cruciate ligament rupture: a meta-analysis. J Orthop Sports Phys Ther.

[5] Buda R, Di Caprio F, Giuriati L, Luciani D, Busacca M, Giannini S (2008). Partial ACL tears augmented with distally inserted hamstring tendons and over-the-top fixation: an MRI evaluation. Knee.

[6] Casagranda BU, Casagranda BC, Maxwell NJ, Kavanagh EC, Towers JD, Shen W, Fu FH (2009). Normal appearance and complications of double-bundle and selective-bundle anterior cruciate ligament reconstructions using optimal MRI techniques. AJR Am J Roentgenol.

[7] Crawford R, Walley G, Bridgman S, Maffulli N (2007). Magnetic resonance imaging versus arthroscopy in the diagnosis of knee pathology, concentrating on meniscal lesions and ACL tears: a systematic review. Br Med Bull.

[8] Duc SR, Zanetti M, Kramer J, Käch KP, Zollikofer CL, Wentz KU (2005). Magnetic resonance imaging of anterior cruciate ligament tears: evaluation of standard orthogonal and tailored paracoronal images. Acta Radiol.

[9] Edwards A, Bull A, Amis AA (2008). The attachments of the anteromedial and posterolateral fibre bundles of the anterior cruciate ligament. Part 2: femoral attachment. Knee Surg Sports Traumatol Arthrosc.

[10] Edwards A, Bull AMJ, Amis AA (2007). The attachments of the anteromedial and posterolateral fibre bundles of the anterior cruciate ligament: part 1: tibial attachment. Knee Surg Sports Traumatol Arthrosc.

[11] Hantes ME, Zachos VC, Liantsis A, Venouziou A, Karantanas AH, Malizos KN (2009). Differences in graft orientation using the transtibial and anteromedial portal technique in anterior cruciate ligament reconstruction: a magnetic resonance imaging study. Knee Surg Sports Traumatol Arthrosc.

[12] Illingworth KD, Hensler D, Working ZM, Macalena JA, Tashman S, Fu FH (2011). A simple evaluation of anterior cruciate ligament femoral tunnel position: the inclination angle and femoral tunnel angle. Am J Sports Med.

[13] Iriuchishima T, Shirakura K, Horaguchi T, Morimoto Y, Fu FH (2011). Full knee extension magnetic resonance imaging for the evaluation of intercondylar roof impingement after anatomical double-bundle anterior cruciate ligament reconstruction. Knee Surg Sports Traumatol Arthrosc.

[14] Katahira K, Yamashita Y, Takahashi M, Otsuka N, Koga Y, Fukumoto T, Nomura K (2001). MR imaging of the anterior cruciate ligament: value of thin slice direct oblique coronal technique. Radiat Med.

[15] Kopf S, Pombo MW, Szczodry M, Irrgang JJ, Fu FH (2011). Size variability of the human anterior cruciate ligament insertion sites. Am J Sports Med.

[16] Muramatsu K, Hachiya Y, Izawa H (2008). Serial evaluation of human anterior cruciate ligament grafts by contrast-enhanced magnetic resonance imaging: comparison of allografts and autografts. Arthroscopy.

[17] Nakayama Y, Shirai Y, Narita T, Mori A, Kobayashi K (2001). The accuracy of MRI in assessing graft integrity after anterior cruciate ligament reconstruction. J Nihon Med Sch.

[18] Pedersen DR, Klocke NF, Thedens DR, Martin JA, Williams GN, Amendola A (2011). Integrating carthage-specific T1rho MRI into knee clinic diagnostic imaging. Iowa Orthop J.

[19] Petersen W, Zantop T (2006). Partial rupture of the anterior cruciate ligament. Arthroscopy.

[20] Schreiber V, van Eck C, Fu FH (2010). Anatomic double-bundle ACL reconstruction. Sports Med Arthrosc.

[21] Shen W, Forsythe B, Ingham SM, Honkamp NJ, Fu FH (2008). Application of the anatomic double-bundle reconstruction concept to revision and augmentation anterior cruciate ligament surgeries. J Bone Joint Surg.

[22] Siebold R, Ellert T, Metz S, Metz J (2008). Femoral insertions of the anteromedial and posterolateral bundles of the anterior cruciate ligament: morphometry and arthroscopic orientation models for double-bundle bone tunnel placement—a cadaver study. Arthroscopy.

[23] Siebold R, Ellert T, Metz S, Metz J (2008). Tibial insertions of the anteromedial and posterolateral bundles of the anterior cruciate ligament: morphometry, arthroscopic landmarks, and orientation model for bone tunnel placement. Arthroscopy.

[24] Siebold R, Fu FH (2008). Assessment and augmentation of symptomatic anteromedial or posterolateral bundle tears of the anterior cruciate ligament. Arthroscopy.

[25] Silva A, Sampaio R, Pinto E (2010). Femoral tunnel enlargement after anatomic ACL reconstruction: a biological problem?. Knee Surg Sports Traumatol Arthrosc.

[26] Sonnery-Cottet B, Lavoie F, Ogassawara R, Scussiato RG, Kidder JF, Chambat P (2010). Selective anteromedial bundle reconstruction in partial ACL tears: a series of 36 patients with mean 24 months follow-up. Knee Surg Sports Traumatol Arthrosc.

[27] Takeda Y, Sato R, Ogawa T, Fujii K, Naruse A (2009). In vivo magnetic resonance imaging measurement of tibiofemoral relation with different knee flexion angles after single- and double-bundle anterior cruciate ligament reconstructions. Arthroscopy.

[28] Van Dyck P, Vanhoenacker FM, Gielen JL, Dossche L, Van Gestel J, Wouters K, Parizel PM (2011). Three tesla magnetic resonance imaging of the anterior cruciate ligament of the knee: can we differentiate complete from partial tears?. Skeletal Radiol.

[29] van Eck C, Lesniak B, Schreiber V, Fu FH (2010). Anatomic single- and double-bundle anterior cruciate ligament reconstruction flowchart. Arthroscopy.

[30] van Eck CF, Schreiber VM, Liu TT, Fu FH (2010). The anatomic approach to primary, revision and augmentation anterior cruciate ligament reconstruction. Knee Surg Sports Traumatol Arthrosc.

[31] Zantop T, Brucker PU, Vidal A, Zelle BA, Fu FH (2007). Intraarticular rupture pattern of the ACL. Clin Orthop Relat Res.

